# Framings of Patient Knowledge and Proximal Technologies in Type 1 Diabetes: A Hermeneutic Review Using Meta‐Narrative Methods

**DOI:** 10.1111/1467-9566.70287

**Published:** 2026-11-01

**Authors:** Nadia Swann, Jackie van Dael, Henriette Langstrup, Eleanor Barry, Trisha Greenhalgh, Chrysanthi Papoutsi

**Affiliations:** ^1^ Nuffield Department of Primary Care University of Oxford Radcliffe Observatory Quarter Oxford UK; ^2^ Centre for Medical Science and Technology Studies (MeST) Institute of Public Health University of Copenhagen Copenhagen Denmark

**Keywords:** hermeneutic review, patient knowledge, type 1 diabetes, wearable technologies

## Abstract

Some people living with type 1 diabetes (T1D) wear glucose sensors and pumps which use algorithms to deliver insulin automatically. These technologies require practical knowledge, yet how patients develop this knowledge remains undertheorised. We conducted a hermeneutic review, informed by meta‐narrative methods, to examine how different research traditions have conceptualised patient knowledge concerning T1D proximal or wearable technologies. Using databases and snowball searches, we classified 74 articles into four research traditions, each carrying assumptions about how patients acquire, develop or enact knowledge. In biomedical literature, patients were typically seen as passive recipients of technology, with knowledge framed as factual or skills‐based. Health psychology literature emphasised individual empowerment through mastery of technical skills. Design and innovation studies treated patient knowledge as an instrument for design. Social science‐informed approaches focused on knowledge as a social process distributed between patients through communities of practice or centred on enacting embodied and situated knowledge. Our review shows how narrow framings risk undermining the richness of T1D knowledge practices with implications for technology development and implementation in the context of diabetes care. We argue for stronger engagement with sociologically informed approaches in future research to attend to the multiplicity of knowledge practices.

## 1 Introduction

Management of type 1 diabetes (T1D) has traditionally required measurement of blood glucose through finger prick testing and multiple daily insulin injections. Proximal or wearable technologies such as insulin pumps and blood glucose sensors (Duke et al. 2018) have been introduced in some high‐income countries. These include closed‐loop technologies which enable automated blood glucose sensing coupled with continuously adjusted insulin delivery using a machine learning artificial intelligence algorithm. Several types of commercial closed‐loop technologies now have regulatory approval across Europe and North America (Anandhakrishnan and Hussain 2024). In 2024, the National Health Service in England became the first health system to provide hybrid closed‐loop technologies free to patients meeting certain criteria (NICE 2023). Prior to the approval of commercial systems, some people living with T1D developed their own automated insulin delivery systems independently of formal providers (Lewis 2019), and some continue to do so.

Closed‐loop systems reduce the need for finger pricking and make automated calculations, reducing mental load. However, substantial work is still needed to maintain the system and make adjustments for food or exercise (Tanenbaum and Commissariat 2023; Cristello Sarteau et al. 2023). How patients acquire and develop knowledge of living with, maintaining and modifying their proximal technologies is important to understand as automated technologies develop. In this review, we examine and synthesise a range of literature on patient knowledge in relation to T1D proximal technologies to surface assumptions underpinning different theorisations and discuss how a sociological lens might contribute to this topic.

### 1.1 Views of Self‐Management

T1D technologies are often welcomed as tools to ameliorate self‐management (Barnard et al. 2017) reflecting a logic focused on acquiring technical skills to increase efficiency and independence (Lorig et al. 1999; Bandura 1978). While good self‐management can encourage improved HbA1c (Lorig et al. 1999; Weise et al. 2024), medical discourses on self‐management give limited attention to potential downsides.

The concept of self‐management has, however, been widely critiqued in sociological literature (Ong et al. 2014; Taylor and Bury 2007), including through burden of treatment theory (C. R. May et al. 2014; C. May 2006) and concepts of responsibilisation (Varey et al. 2021). Sociological literature frames self‐managing a chronic health condition as complex, relentless and time‐consuming (Campbell et al. 2003), requiring practical, cognitive and socio‐emotional ‘work’ (Hinder and Greenhalgh 2012; Corbin and Strauss 1985). This literature foregrounds practical knowing (Polanyi 2009; Bourdieu 1972) acquired through experience and embodiment (Brown et al. 2004), reflecting Merleau‐Ponty's understanding of a knowing body as a fundamental way of knowing about the world (Merleau‐Ponty 2013). Mol and Law apply such concepts of knowing to the practices of living with diabetes technologies, exploring embodied action or how we *do* bodies (Mol and Law 2004) in the context of hypoglycaemia. Such theorisations may advance our learning concerning patient knowledge practices in the context of proximal diabetes technologies.

### 1.2 Technologies‐in‐Use

In design disciplines, patient experiences are often viewed as resource for design improvements; a factual aid to innovation or to maintain safety. This view draws on lead user approaches to innovation (von Hippel 2005), foregrounding a motivated and knowledgeable patient as user, or takes a quantitative approach to user safety by drawing on research traditions of human factors and ergonomics (Rasmussen 1982).

Social science scholars have explored how the construction of patient knowledge regarding T1D proximal technologies is linked to underlying assumptions which critique forms of technological determinism, portraying technologies as socially neutral, as drivers of social change or as leading to dystopian outcomes (Wyatt 2023). Such narratives risk overlooking the rich situatedness of living with a long‐term health condition, where technology use is co‐constituted through everyday practices (Papoutsi et al. 2021). Sociologically framed studies relating to T1D focus on how technologies are reconfiguring self‐management and self‐care practices, highlighting mutual constitution of devices, bodies and users (Berk 2018; Mialet 2022). Such framings have engaged with a broader, situated view of medical technologies across healthcare contexts, exploring technologies as an ensemble of people and technologies (Timmermans and Berg 2003). Comparing and contrasting views of patient knowledge across a range of research traditions from biomedical to sociological may help to interrogate roles assigned to patients and clinicians (Brown et al. 2004), which is important given clinical power to provide, refuse or withdraw access to T1D technologies (Farrington et al. 2020; Lawton et al. 2016; Tanenbaum et al. 2017; Ardissone 2021; Addala et al. 2021; Øversveen 2021). Sociological framings may develop richer understandings of what it means to *know* technology in T1D, given the rapid pace of change.

### 1.3 Research Questions

To explore different framings of patient knowledge in the literature on T1D proximal technologies, we asked the following questions:

- What research traditions have explored patient knowledge in the context of T1D proximal technologies?
- How have these research traditions presented their theorisation of patient knowledge of proximal T1D technologies over time?
- What can we learn from findings across these research traditions and what are priorities for further research?

In this review, we have used proximal technologies to mean insulin pumps, sensors, related software and algorithms (Duke et al. 2018).

## 2 Methods

We carried out a hermeneutic review using meta‐narrative methods. A hermeneutic approach (Boell and Cecez‐Kecmanovic 2014) emphasises deep, iterative engagement with a body of literature for gradual development of understanding, surfacing meaning through interpretation and critical assessment. Meta‐narrative methods prioritise theory‐driven interpretative techniques to make sense of heterogeneous bodies of literature that approach the same problem from different perspectives over time (Wong et al. 2014).

We designed our search strategy through an iterative process, carrying out a keyword search on type 1 diabetes and terms related to knowledge, experience or self‐care in the context of T1D proximal technologies across Medline (3/10/23), Social Sciences Citation Index (4/10/23), CINAHL (5/10/23) and PsycINFO (5/10/23) databases (see Supporting Information S1 for search terms). The lead author reviewed 5358 titles and 324 abstracts to determine if they met the inclusion criteria, removing duplicates and reviewing 115 full text articles to select 58 full text articles and 16 additional articles from snowball/forward and backward citations tracking to produce 74 articles for full analysis.

Adopting a hermeneutic approach (Boell and Cecez‐Kecmanovic 2014) enabled us to trace narrative development in relevant traditions by hand‐searching reference lists of papers, tracking backward and forward citations and asking for expert recommendations. To extract the 74 articles for full text analysis, we used Greenhalgh et al.’s overriding question: ‘is this paper likely to *add meaning* to our emerging overview of the field?’ (2017). To explore meaning in these articles, we employed a reflexive approach using configurative logic. This involves consideration of multiple interdependencies, a common approach in hermeneutic review, which is suitable for exploring heterogeneous bodies of literature (Greenhalgh 2026 (in press); Greenhalgh et al. 2018), using judgement alongside topic and background expertise (Lorenc et al. 2016; Briscoe et al. 2025).

To ensure a quality contribution in accordance with publication standards for articles using meta‐narrative methods, we followed the six principles of meta‐narrative review (pragmatism, pluralism, temporality, contestation, reflexivity and peer review) (Wong et al. 2014; Noyes et al. 2025). A summary table of the application of these principles to our review is available in the Supporting Information S1.

Informed by other hermeneutic and meta‐narrative reviews (Greenhalgh et al. 2005, 49; Papoutsi et al. 2021; Greenhalgh, Robert, et al. 2005, 417), the lead author used an adapted data extraction form (Greenhalgh et al. 2005, 232) to assess each article in the context of research tradition, theoretical basis, study design, validity of methods, nature and strengths of findings and conclusions and relevance of backward and forward citations (Briscoe et al. 2025). Working iteratively, the lead author looked for conceptual similarities and differences related to patient knowledge and its theorisation and tracked theorisation sources back to seminal papers of origin. A second member of the research team carried out close reading of selected papers to contribute to the categorisation process. Categorisations were discussed in depth across the research team who decided on their inclusion and allocation by majority consensus.

Our analysis focused on how storylines of main research traditions have unfolded over time (Wong et al. 2014), tracing how influences of ‘seminal theoretical and empirical work’ (Greenhalgh, Robert, et al. 2005, 417) have shaped the literature. We gave consideration to Kuhnian defining characteristics of scientific traditions (Kuhn 2012), including the historical concept on which traditions are based, theoretical frameworks which influence research questions and ‘preferred methods’ (Greenhalgh et al. 2005, 49) (see Table 1 in Section 3 below). As part of our progressive focusing, we concentrated our detailed analysis on articles from searches published in the 10‐year period from 2013 to 2023, as it emerged that this was when relevant publications on newer T1D proximal technologies gained momentum.

We follow the Cochrane‐Campbell Handbook approach to reporting meta‐narrative methods in this review (Noyes et al. 2025), presenting the ‘story’ of each meta‐narrative in our findings, exploring how ways of viewing patient knowledge of T1D technologies were informed by these traditions and synthesising commonalities and tensions across traditions, following meta‐narrative principles of contestation (Greenhalgh et al. 2005, 44).

## 3 Findings

There were 74 articles included for full review (see Figure 1 below). Articles were in English, predominantly from 2013 onwards and included 16 UK and 27 US focused articles, with 5 articles from Australia, 6 from New Zealand, 3 from Sweden, 2 from Norway, 2 from Italy, 1 from Spain, 1 from Switzerland, 2 from France, 3 from Germany, 1 from Denmark, 1 from the Netherlands, 1 from India, 1 from China and 2 multi‐national surveys. The dataset included qualitative research studies (*n* = 46) such as ethnographies and studies using semi‐structured interviews, literature reviews using systematic or narrative synthesis approaches (*n* = 12), quantitative survey studies (*n* = 11), mixed methods studies (*n* = 4) and a research feature article (*n* = 1). Participants were usually patients and/or caregivers. Sample sizes ranged from one to six participants in qualitative studies (Berk 2018; Marshall et al. 2019) to 1461 respondents in a multi‐jurisdictional online survey (Lee et al. 2017). A table of study characteristics is provided in the Supporting Information S1. Below we set out the four main meta‐narratives we found in the literature on patient knowledge and T1D proximal technologies (and we summarise these in Table 1).

**FIGURE 1:**
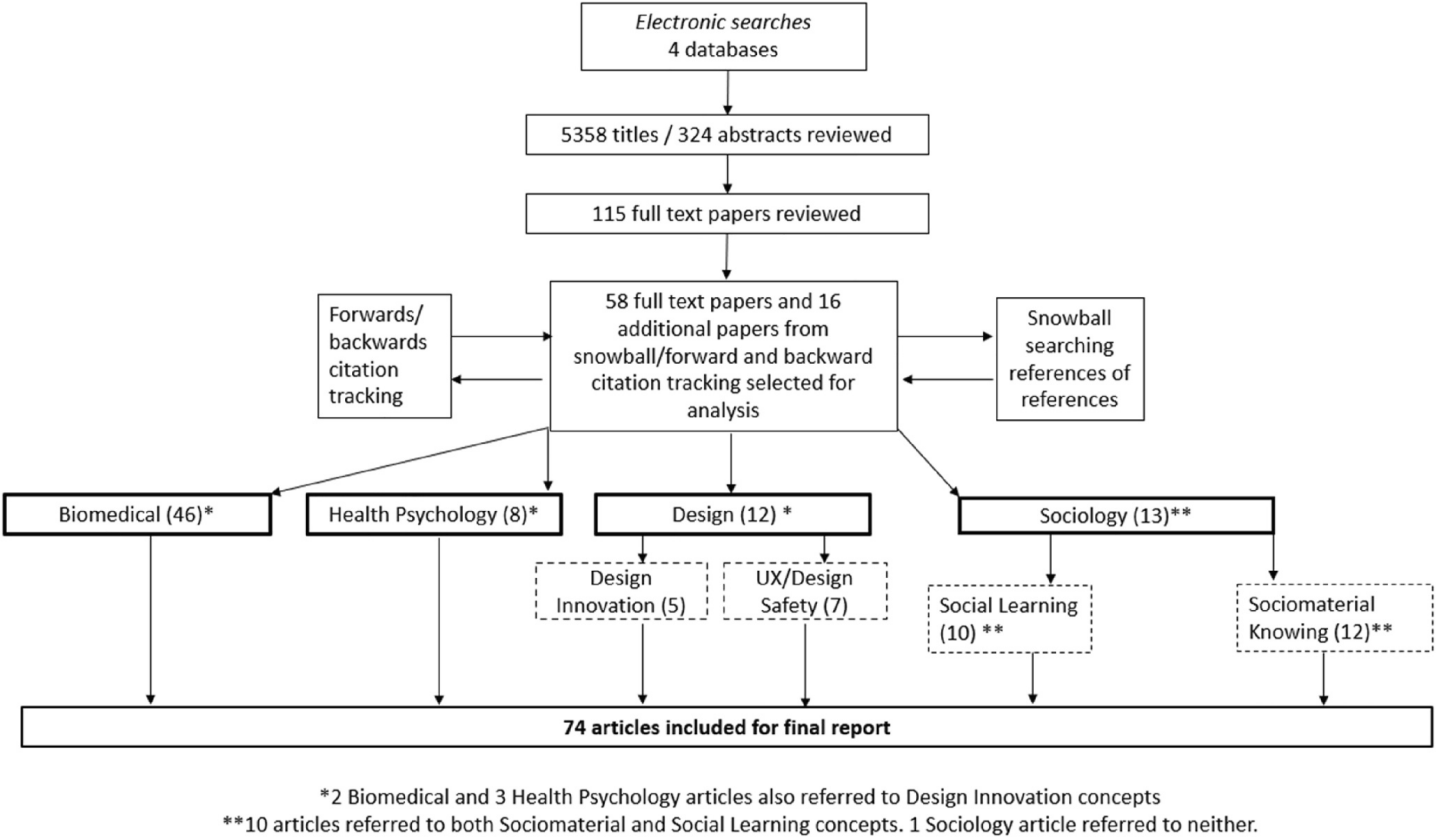
Schematic of search process and articles identified.

**TABLE 1:** Summary of findings: Meta‐narrative comparison table based on approach taken by Greenhalgh et al. (2005, 46) to Kuhn's defining characteristics of research traditions (2012).

Meta‐narrative groupings:	1 Patient knowledge as factual or skills‐based, transferred by clinicians	2 Patient knowledge as self‐mastery to reach individual empowerment	3 Patient knowledge as an instrument for designing better technologies and systems		4 Patient knowledge as relational and sociomaterial	
			Patient knowledge as sources of innovation	Patient knowledge as operational challenge/risk	Patient knowledge as social process	Patient knowledge as situated practice
Patient knowledge viewed as:	Patients are seen as relatively passive recipients of technology, with a focus on attitudes or experiences.	Patients are seen as individuals empowered through technologies.	Patients are seen as technology users.	Patients are seen as the human factor or risk factor in technical systems.	Patients are seen as relational actors, who share and learn from each other and possess different forms of expertise in community.	Patients are seen as situated actors.
	Patient knowledge is mainly conceptualised as factual or skills‐based knowledge, transferred to compliant patients by clinicians.	Patient knowledge through empowerment is dependent on the acquisition of knowledge often conceptualised as the individual mastery of skills and attained through patient education.	Patient knowledge is often conceptualised as user experience, forming a source of feedback for innovation, through inputting into interface design or system features.	Patient knowledge is conceptualised as in need of control, where individual agency and workarounds may contribute to safety risks.	Patient knowledge (e.g., experiential, embodied) is often approached as challenging the epistemic authority of biomedicine.	Patient knowledge is conceptualised as a practical knowing‐in‐action, accomplished through human, bodily, social and technological entanglements as part of daily living.
Dominant discipline	Biomedicine	Health psychology	Design and innovation studies	Human factors and ergonomics	Sociology	Sociology; science and technology studies
Example research question	What are the experiences of trial participants in using hybrid closed loop? (Rankin et al. 2022)	What types of uncertainty/empowerment do patients experience in using T1D technologies and how do they manage/respond? (Perez et al. 2019)	What is the evolution of patient driven innovations from open‐source systems? (Ng et al. 2020)	How do human factors affect performance or safety of devices? (Beneyto et al. 2021)	How are different forms of participation explored among those using open‐source systems? (Jansky and Langstrup 2023)	How does pump‐human entanglement configure responsibility in the lived experience of agency? (Berk 2018)
Examples of theories used	Explicit theory rarely used	Technology acceptance model (Holden and Karsh 2010) or theory of planned behaviour (Ajzen 1985)	Theories of user innovation (von Hippel 2005) or innovation communities (von Krogh and von Hippel 2006)	Human factors and systems theory (IEA 2024)	Theories of communities of practices (Lave and Wenger 1991) or epistemic communities (Akrich 2010)	Theories on knowing as being (Haraway 1991) or doing (Mol 2000)
Preferred methods/Instruments	Randomised controlled trials; sometimes combined with secondary qualitative research (e.g., surveys, focus groups, interviews)	Applied qualitative research, typically involving semi‐structured interviews or surveys	User focus groups	Metrics focused methods (e.g., sensor measurements) or cultural probes in technology design	Interviews; ethnographic methods	Interviews; ethnographic methods

### 3.1 Biomedical View—Patient Knowledge as Factual or Skills‐Based and Transferred by Clinicians

Forty‐six of the 74 articles were framed biomedically. Researchers focused on patient experiences of T1D proximal technologies including barriers and enablers to technology use (see Table 1) (Tanenbaum et al. 2017; Cristello Sarteau et al. 2023; Huhndt et al. 2022; O'Donnell et al. 2023). These studies reflected the epistemological paradigms of seminal biomedical research such as a landmark randomised controlled trial on the use of pump technologies to reduce glycated haemoglobin (HbA1c) and complications (Nathan et al. 1993) or 15 ‘clinical experiments in subjects with type 1 diabetes’ of an early trial version of a basic closed‐loop system called a ‘non‐linear model predictive controller’ to control medication through an insulin pump automatically (Hovorka et al. 2004).

Biomedically framed studies often used the word subject or patient, rather than referring to a person living with T1D. Secondary studies in technology trial populations focused on exploring patient experiences, often referencing HbA1c or hypoglycaemia events as a benchmark measurement (Lakshman et al. 2024; Burnside et al. 2023; Kubilay et al. 2023; Hood et al. 2022) There was little reference to the term patient knowledge; studies focused on technology attitudes (Hood et al. 2022; Tsai et al. 2022) and provided thematic description of what patients valued or found challenging about adjusting to new technology (Rankin et al. 2022; Cristello Sarteau et al. 2023; Kubilay et al. 2023; Brew‐Sam et al. 2021; Hood et al. 2022).

The silhouette of the Parsonian patient emerged, one depicted as the passive subject of a technology trial or experiment, in a manner resonant of Parsons' 1950s description of compliant patient and expert doctor (Parsons 1991). Patient knowledge was often presented as factual and skills‐based, with articles focused on deficits or gaps in people's abilities to use technologies. Education was recommended for patients and/or clinicians as a solution to barriers to technology use (Crabtree et al. 2022). Technology was presented as driving improvements in glycaemic control, quality of life (often a secondary measure) and as motivation for self‐management (Lakshman et al. 2024; Barnard et al. 2017). Potential for improvement was often evaluated through ‘surrogate’ outcomes such as HbA1c, with only one study recognising that this limited ability to explore what was ‘meaningful’ to patients (Natale et al. 2023).

Biomedically framed articles sometimes viewed patient knowledge in terms of risks that patients were prepared to take to avail themselves of new technologies. In one UK survey of 317 clinicians, the five multiple‐choice answer options framed open‐source closed‐loop as ranging from ‘dangerous’ and ‘risky in the wrong hands’ to ‘safer because only used by expert individuals’ (Crabtree et al. 2022), reflecting a view of patient knowledge informed by biomedical perceptions of risk. Articles described how patients sometimes hid their use of open‐source closed‐loop, with clinicians describing this as ‘unfortunate’, calling for more ‘openness’ between patient and clinician (Raimond et al. 2022; Crabtree et al. 2022).

Clinical assumptions about patient knowledge were also visible in studies which reported clinicians expressing surprise in interviews when commenting on patients' unexpected levels of technological knowledge and ability (Farrington et al. 2020; Addala et al. 2021; Lawton et al. 2016, 2020; Howe et al. 2023; Kubilay et al. 2023), supporting our hypothesis that there may be differences between patients' knowledge and clinicians' views of such knowledge.

While biomedical research has played a critical role in supporting the pace of T1D technology development through trials testing safety, efficiency and understanding patient experiences, sociological research traditions may explore and surface different ways of knowing in the context of T1D proximal technologies which enrich our understanding.

### 3.2 Health Psychology View—Patient Knowledge as Self‐Mastery to Reach Individual Empowerment

A second meta‐narrative theorised patient experiences through a health psychology lens, with a focus on individual empowerment. Like biomedical framings described above, research questions focused on patient attitudes towards technology, exploring what patients know through surveys or semi‐structured interviews. Study findings included quality of life measurement scales, quantifying the patient experience or made recommendations of education to overcome barriers to uptake. Articles in this category reflected epistemological paradigms prevalent in health psychology literature around the role of technology, such as the Technology Acceptance Model (Davis 1989) predicting technology uptake though its perceived usefulness, the Health Beliefs Model which theorises how people will take preventative health action when benefits outweigh costs (Rosenstock 1974) or the Theory of Planned Behaviour in considering variables which might influence human behaviour (Ajzen 1985).

Health psychology articles foregrounded the individual patient as rational agent of change (in contrast to the biomedical meta‐narrative which placed technology at the centre) and patient knowledge as factual knowledge constituting mastery of skills. Health psychology framings were not focused on broader social complexities of what it means to self‐manage a complex, chronic medical condition in the context of technologies. Articles reflected concepts of patient empowerment from diabetes education (Funnell et al. 1991) referencing self‐efficacy, for example, to explore whether sensors have an empowering effect on patients (François et al. 2023). Other studies used approaches such as uncertainty management theorisation (Perez et al. 2019), signposting technological knowledge as a way out of uncertainty.

Articles using psychology framings to explain what influences behaviour change or individual patient empowerment prescribed solutions such as psychoeducational training for improved self‐management, or suggested more research into utilising technology as ‘leverage’ to ‘enhance patient education’ (Gonder‐Frederick et al. 2016). Such approaches did not always create space to explore less examined concepts, such as sociological concepts of technology or education contributing to increased responsibility or burden of long‐term self‐care (C. R. May et al. 2014).

### 3.3 Design View—Patient Knowledge as Resource and Instrument for Design of Better Technologies and Systems

Factual and skills‐based knowledge was also a focus of articles emerging from design traditions, which approached patient knowledge either as: (1) a useful means of innovation; or (2) as requiring careful management to maintain safety (see Table 1). Methods were more varied and included focus groups or workshops (McCarthy et al. 2021; Suttiratana et al. 2022), cultural probes such as photovoice (Morrow et al. 2022) and methods of letter writing (McCarthy et al. 2021). Design studies primarily centred on exploring user experience to enhance system features, foregrounding knowledge as an input into design or as data points for enhancing safety.

This design framing depicts a view of a highly motivated and knowledgeable patient as designer and innovator. This small group of articles included analysis of social media and website content (Ng et al. 2020), literature reviews (Reinius et al. 2022) and research feature articles (DeMonaco et al. 2019) related to patient‐driven technologies. Their epistemological origins lie with literature on open innovation as social disruptor where lead users create new technologies and freely share their learning in innovation communities to democratise and rival commercial innovation (Chesbrough 2003; von Hippel 2005). Articles belonging to this meta‐narrative cited Dana Lewis, her partner Scott Leibrand and the community behind the invention of open‐source closed‐loop (Lewis 2019, DeMonaco et al. 2019) or alluded to ‘D‐Dads’ such as John Costik or Ben West, part of the parent T1D community who invented free open‐source software to access sensor data (Ng et al. 2020), as examples of knowledge creating and sharing by lead users.

Some articles, underpinned by the design meta‐narrative, focused on eliciting user experience towards safety improvements. These articles have their origins in human factors or ergonomics traditions, influenced by aviation and engineering research (Shorrock and Williams 2016). They included quantitative analysis of sensor data (Staszak et al. 2021) and systems safety analysis (Beneyto et al. 2021) along with qualitative studies of user experience (Suttiratana et al. 2022). Patient knowledge was positioned as an ‘operational’ challenge in T1D technologies (Suttiratana et al. 2022) or a ‘disturbance’ caused by the human ‘fourth component’ of the system after sensor, pump and algorithm (Beneyto et al. 2021). Patients' knowledge of workarounds introduced ‘faults’ into the system, such as a person eating food without ‘notifying the controller’ in hybrid closed‐loop (Beneyto et al. 2021). Patient input required ‘vigilance’ (Nefs 2022) to reduce risk and design safer technologies, with psychosocial education seen as a solution, similar to biomedical and psychological framings above (Nefs 2022).

In summary, articles in this meta‐narrative viewed knowledge as input or variable, paying less attention to the human being behind that variable and the multiple, contextual practices of knowing. Exploring the sociological entanglement of human and non‐human actors might illuminate situated complexities of knowing in richer ways to influence design and safety.

### 3.4 Sociological View—Patient Knowledge as Relational and Sociomaterial

A small body of literature offered sociologically‐informed theorisations of patient knowledge of T1D technologies from the perspectives of: (1) collective social learning process and (2) situated sociomaterial practices (see Table 1). Articles used a variety of research methods, encompassing autoethnography (Berk 2018), ethnographic interviews (Ardissone 2021; Kaziunas et al. 2017; Jansky 2023), assemblage ethnography (Jansky and Langstrup 2023), observations (Kaziunas et al. 2018; Danesi et al. 2020), show‐and‐tell (Mathieu‐Fritz and Guillot 2017) and case studies (Ardissone 2022). Research questions focused more on *how* patients know (socially or through sociomaterial practice) rather than *what* they know (see Table 1).

Stories of collaborative, social learning are reflected in this framing. These were informed by theorisations of lay expertise or expert knowledge through shared and collective lived experience, including in terms of epistemic communities challenging the clinical status quo (Borkman 1976; Epstein 1995; Wilcox 2010; Akrich 2010). Articles further explored knowledge development as sociocultural in communities of practice (Lave and Wenger 1991, 29–31).

In one study, a group of software programmers whose children lived with T1D learnt to ‘hack’ a sensor, ‘liberating’ the child's blood glucose measurements to see these numbers remotely in real time (Kaziunas et al. 2017). This became Nightscout, an open‐source system for reading a sensor remotely. The work involved in this knowledge acquisition forms a hard‐earned apprenticeship. A ‘sleep‐deprived’ father described how the open‐source community provided him with resources and ‘install parties’, face‐to‐face groups to learn from experienced users and an online knowledge‐sharing group with thousands of members (Kaziunas et al. 2017). In another story, open‐source developers taught a grandmother to use a soldering iron to construct closed‐loop hardware for her grandchild (Kaziunas et al. 2018). Learning was viewed as a shared social experience, in the ‘complex anxious‐togetherness’ of communities developing practices outside clinical contexts (Kaziunas et al. 2017). People building the first open‐source closed‐loop were described by the authors as carrying out knowledge sharing through data in ‘secretive, risky and ethically fraught activity’ which ‘required careful negotiation of what was safe or risky to share’ (Kaziunas et al. 2017).

Patients self‐experimented and shared code and knowledge (Lewis 2019) resulting in the formation of activist communities. For example, knowledge of Nightscout open‐source coding enabled users to engage with policy and regulation for legal approval of parts of their system and to push for approval of commercial systems more quickly, using the online community #*WeAreNotWaiting* hashtag, in an example of a collective patient‐led advocacy community (Kaziunas et al. 2018). Articles in the sociological meta‐narrative linked the political with the experiential, describing how Dana Lewis translated lived expertise using ‘classic clinical language’ to show how their open‐source algorithm met ‘unmet needs’ (Gottlieb and Cluck 2018) or Ben West of Nightscout describing to regulatory authorities how not having access to bodily data was doing him medical ‘harm’ (Gottlieb and Cluck 2018). Rather than noting that patients hiding their use of these systems was unfortunate or lacking transparency for clinical purposes (Raimond et al. 2022; Crabtree et al. 2022), these articles explored how and why the patient community learnt to hesitate to disclose their knowledge or hide their use of open‐source systems, for fear of consequences; that is, that clinicians may perceive them as non‐compliant and/or exert gatekeeping powers over technologies (Kaziunas et al. 2018; Gottlieb and Cluck 2018).

Some articles drew on what we term sociomaterial knowing as a way of conceptualising patient knowledge in the context of T1D proximal technologies. Research questions focused on understanding facets of co‐constituted human, bodily, social and technological entanglements (Berk 2018; Mialet 2022) (see Table 1). These articles critiqued techno‐utopian discourses in knowledge and use of T1D proximal technologies (Ardissone 2021; Jansky 2023; Mathieu‐Fritz and Guillot 2017). Articles in this sub‐group drew strongly on science and technology studies, including Haraway's descriptions of cyborgs as an enmeshment in the blurring of human and machine (Haraway 1991). Mol's landmark study on the use of at‐home glucose monitoring technology (Mol 2000), concepts of ‘tinkering’ (Mol et al. 2010) and knowing‐in‐practice (Mol and Law 2004) have informed an understanding of adaptations and workarounds to make T1D technologies work in situated contexts, in what Pols has described as ‘practical knowing in action’ (Pols 2014). Articles explored how knowing may develop reflexively through entanglement with bodily inserted sensors (Jansky and Langstrup 2023; Mialet 2022), as ‘companions’ or cohabitors who enter flesh literally, in a kind of ‘kinship’ (Ardissone 2021). Studies focused on how such knowing can also involve pain and discomfort, describing the work involved in ‘extracting data’ through inserting sensors into the skin as empowerment at ‘high cost’ (Kaziunas et al. 2018). We also see how the knowing practices involved in the context of T1D technologies may create ‘dissonance’ between ‘bulky’ uncomfortable tubed insulin pumps and patients' ‘desire to live an untroubled life’ (Ardissone 2021). Patient knowledge in these articles often formed part of an assemblage and/or was experienced as ‘multiple’ (Kaziunas et al. 2017). Living with an insulin pump was framed as ‘embodied cyborg subjectivity’ (Berk 2018). Devices were viewed as self‐sensory (Gottlieb and Cluck 2018; Danesi et al. 2020; Mialet 2022), reflecting Haraway's concepts of sensing machines as ‘prosthetic devices, intimate components, friendly selves’ (Haraway 1991, 178), albeit ones which sometimes require ‘elaborative tinkering’ (Jansky 2023), drawing on Mol's concept of tinkering as ‘creative calibrating of elements’ to meld with the situated environment (Mol 2006).

In articles on open‐source closed‐loop technologies, expression of learning processes reflected notions of ‘illegitimate fusions’ (Haraway 1991, 176) between human, hardware and unregulated algorithmic coding. Descriptions of cyborg people who rewrite the ‘texts of their bodies and societies’ in order to survive is significant for its prediction of the way in which open‐source closed‐loop communities have had to rewrite the compliant patient script in order to access technology that enables them to survive (Haraway 1991, 177). Knowledge gained through experimentation and risk‐taking was explored (Jansky 2023; Jansky and Langstrup 2023; Kaziunas et al. 2018), reflecting Mol's concepts of attentive experimentation (Mol 2006, 2008). Articles also reflected Haraway's descriptions of technologies as being simultaneously ‘friendly selves’ and ‘monsters’ (Haraway 1991, 178) in their use of language, describing the relationship as one of ‘frightening intimacy’, being both fascinating and fearful (Kaziunas et al. 2018).

Some articles in this sociological meta‐narrative alluded to power dynamics, including how patients' use of T1D technologies involves assumptions about good and bad patients or behaviour (Gottlieb and Cluck 2018) and drawing on Foucauldian disciplining practices in the clinical oversight of patient self‐management (Foucault 1977). One study from Italy described the dynamic around technology allocation as a ‘subtle interactional tug of war’ (Ardissone 2021) drawing on concepts of ‘covert conflict over power and control’ (Greenfield et al. 2012). Another article from Norway considered the ‘complex and fraught’ relationship between ‘medical technology and medical power’ (Øversveen 2021) in determining whether a patient was deemed ‘worthy’ to receive a sensor, a tool referred to as a ‘reward’ (Øversveen 2021). This article was something of an outlier in that it focused less on sociomaterial theorisation and more on situated power dynamics. Other articles explored responses to power imbalances as forms of resistance or related how open‐source has evolved, in part, from power shifts between individual and authority (Gottleib and Cluck 2018; Kaziunas et al. 2018). Phenomena of surveillance were described in patients refusing to share their bodily sensor or pump data with clinicians for fear of judgement (Mathieu Fritz and Guillot 2017).

Theorisation also included a lens of distributed cognition (Goodwin 1995; Hutchins 1995) combined with phenomenology, exploring how patient knowledge is distributed through an environment of ‘humans, artefacts and machines’ (Mialet 2022). However, theories of embodied cognition alone may not explain the cognitive work required in interacting with surroundings to calculate, for example, how much energy climbing a flight of stairs will take and whether to adjust machine settings (Mialet 2022). This is knowledge co‐constituted between patient and machine.

### 3.5 Similarities and Differences Across Research Traditions

Having reviewed the meta‐narrative ‘stories’ of our topic through the lens of distinct research traditions, we set out below a brief synthesis of example similarities and differences in research tradition.

Relevant to this synthesis, 2 biomedical (Ng et al. 2020; Kubilay et al. 2023) and 3 health psychology articles (Gonder‐Frederick et al. 2016; Gonder‐Frederick et al. 2011; Tanenbaum et al. 2020) contained hybrid concepts which overlapped with design innovation (see Figure 1). Ten of the 13 sociological articles were framed from the perspective of social learning but often also drew on sociomaterial lenses. One sociology article focused on allocation of limited resources of new sensors and pumps using a health inequalities lens (Øversveen 2021). These examples show how our groupings, while based on predominant characteristics, are not rigid or immutable but are created iteratively by the research team as ways to frame the views of people living with T1D and their technologies.

In an example of similarities, in the meta‐narrative stories outlined in our findings, a theme of individual patient ‘compliance’ could be traced across biomedical and safety design traditions, where patients were often depicted as passive ‘subject’ (or at best co‐operative) individual device ‘user’ (Staszak et al. 2021; Beneyto et al. 2021). Health psychology and design innovation framed user compliance more as empowerment (François et al. 2023; DeMonaco et al. 2019). The approach taken in the sociological literature contrasts these framings through exploring compliance in terms of power dynamics, whether it be patient activist practices of conforming through using medical language in order to be taken seriously and bring about change (Gottlieb and Cluck 2018) or through the non‐compliance of covert information withholding about self‐coded adaptations to technology (Kaziunas et al. 2018; Gottlieb and Cluck 2018).

Across biomedical health psychology and design framings, we also traced narratives of ‘competencies’; knowledge in terms of technical competence and the barriers and enablers which patients need to overcome to be able to use devices for optimal clinical outcomes (Cristello Sarteau et al. 2023; François et al. 2023; Suttiratana et al. 2022).

In contrast with the sociological literature, few articles in biomedical, health psychology or design literature paid attention to the concept of patients as ‘activists’; in the sense of being actively engaged in such knowledge generation, other than innovation design which sometimes foregrounded exceptional lead users developing open‐source T1D technologies (DeMonaco et al. 2019). Biomedical and health psychology framings presented a consistent theme of knowledge types as hierarchical, with biomedical knowledge at the top and experiential knowledge at the bottom or viewed as deficit, sometimes leading to clinical ‘surprise’ on discovering patients' knowledge (Farrington et al. 2020; Lawton et al. 2016). This view was often coupled with a presumption of knowledge transfer from clinician to patient with education of the patient presented as solution to the knowledge deficit problem (Gonder‐Frederick et al. 2016; Crabtree et al. 2022).

In sharp contrast to themes of individual compliance, passive use and one‐way knowledge transference, sociologically informed research identified a more nuanced and relational understanding of patient knowledge and themes of patients as important epistemic actors (Jansky and Langstrup 2023). This approach takes our understanding of patient knowledge beyond a limited focus on the individual as a container to be filled with technical skills or motivation or as an ‘exceptional’ lead user, to foreground the concept of knowing as an ongoing practice, accomplished through interactions among people, communities, healthcare contexts, algorithms and devices. In contrast to approaches to knowledge in biomedical framings particularly, knowledge in this framing is social and performative, bringing the situated and complex practices of knowing (rather than knowledge) to the fore (Kaziunas et al. 2018; Kaziunas et al. 2017). These sociologically framed examples show us how patients ‘*know*’ with and through technologies.

## 4 Discussion

Exploring individual meta‐narratives and synthesising example similarities and differences show us how more narrowly framed studies of patient knowledge in biomedical, health psychology and design may become acutely insufficient in living with T1D proximal technologies. Such understandings are developed more fully in the richer nuances of the sociological studies reviewed. As suggested above, sociological literature may describe the kind of patient knowledge explored as ‘knowing’ rather than knowledge, reflected in the example of self‐help groups where experiential knowledge is explored as ‘truth learnt from personal experience with a phenomenon’ (Borkman 1976). Nicolini explores knowing as a ‘nexus of interconnected practices’ rather than viewing knowledge as a substance that we employ (2011). Such interconnectedness forms part of the development of ‘practical knowing in action’ referred to above and helping us explore the differences between the ‘what’ and ‘how’ of knowing (Pols 2014) in the context of T1D proximal technologies.

An emphasis on the sociological framing of knowing opens up space for a plurality of experiences emerging from the entanglement of human and non‐human actors in an exploration that biomedical, psychology or design framings struggle to encompass. The technical, recursive and methodological knowledge practices that the *#WeAreNotWaiting* open‐source community (Braune et al. 2021) employ to build and code their own closed‐loop system, for example, can only be understood through rich sociologically framed accounts (Jansky 2023), practices that a biomedical framing may reduce to ‘risky’ in a questionnaire (Crabtree et al. 2022). Increased access to and use of advanced T1D technologies provides opportunity for sociological inquiry into how patients ‘*know’* with and through technologies outside of formal biomedical knowledge hierarchies or clinician mediated patient education. With the promise of more types of closed‐loop advanced technologies for T1D (Royston et al. 2025) and other chronic diseases (e.g., Parkinsons disease and Epilepsy) in the future, further research should include more focused, sociologically framed studies of how knowing and knowledge take shape among those who live with T1D and other chronic diseases and their intimate technologies.

Future research encompassing sociologically‐informed accounts viewing self‐care as a constellation of technical tools, knowledge practices and physical and emotional work (Campbell et al. 2003; Hinder and Greenhalgh 2012; Kingod and Cleal 2019) may contribute rich insights. Translating some of these insights for other disciplines may also help interrogate implicit (or explicit) assumptions about technologies, users and use‐situations, departing from deterministic framings and using sociological theory to conceptualise devices, bodies and users as mutually constituted. Sociologically framed approaches open up opportunities to redress epistemic injustice (Fricker 2007) in dominant paradigms that prioritise individualist, skills‐focused framings of knowledge. For example, a perception of knowledge deficit in a biomedical framework (such as a patient manually overriding a closed‐loop algorithm by bolusing for non‐existent or ‘ghost’ carbohydrates to lower high blood glucose more quickly) might alternatively be explored sociologically through knowing in practice, exploring how the person knows their bodily reactions to a particular algorithm in particular contexts. Microdosing for ghost carbohydrates may temporarily alleviate high glucose levels, but it requires personalised bodily knowledge, care and cautious experimentation—concepts that may benefit from sociological exploration for richer understanding.

Sociologically framed research focused on developing our understanding of situated knowledge practices has the potential to lead to better development of technology‐supported T1D care, in informing a better understanding of the ongoing work required to generate and maintain knowledge practices and what this means for how (self‐)care and service provision become configured. For example, a person with regularly high glucose levels eating a fast‐food lunch at work who then becomes hypoglycaemic when driving home may look like someone who has ‘behavioural deficits’ or ‘poor’ eating practices from a clinical perspective. Sociological exploration of situated knowledge and practices may surface that the person is also working in a toxic office environment with stressful afternoon deadlines. The person knows that this stress is contributing to temporary and stubborn high glucose levels (Wiesli et al. 2005) which the algorithm is unable to ‘see’, so the system continues to deliver repeatedly higher levels of background insulin, contributing to the severity of the hypoglycaemia when the person leaves the office. An understanding of this would enable care and support in this example to be focused on a collaborative discussion regarding insulin dosing for food and stress, rather than a narrower focus on ‘bad’ eating practices.

Better consideration of power dynamics combined with sociological theorisation may help to explore patient‐led healthcare technologies developed outside of medical contexts, such as in the example of how competing knowledge may affect power balances between clinician and patient in using pumps and sensors (Ardissone 2021). A sociologically framed approach may explore assumptions where a patient may be ‘ruled in’ for access or ‘ruled out’ on the basis of whether they are ‘especially deserving of help’ (Hughes and Griffiths 1997). As described in our findings, clinical staff acknowledge sometimes relying on their preconceptions of patients (Lawton et al. 2020) or tacit and informal criteria to decide who is suitable for such technologies (Farrington et al. 2020; Lawton et al. 2016, 2020). Sociological studies of moral evaluation of patients for treatments (Glaser and Strauss 1964), assessed through perceived social worth (Wamsiedel 2018) may have relevance to contexts of T1D technology allocation. Greater use of social science framings is needed to inform other disciplines in this topic area and enable more productive understandings.

### 4.1 Strengths and Limitations

In this review, we contend with a significant body of literature across disciplines. We retrieved this using carefully planned search processes, including iterative searching and progressive focusing. We used the RAMESES quality standards to help us decide on a good quality contribution in each tradition and taking a pragmatic approach to sense‐making (Greenhalgh, Robert, et al. 2005). A hermeneutic approach allowed us to focus on interpreting (rather than purely describing) the assumptions underpinning the research in the articles analysed in a rich, nuanced manner. Additional strengths lie in the use of meta‐narrative principles to ensure that we viewed the topic area from multiple angles including to trace the epistemological foundations informing each meta‐narrative. The lead author's background expertise (Barbour 2001) as an academic researcher, family member of people living with T1D and director of a medical research charity focused on T1D—alongside the breadth and depth of the research team's expertise across the social sciences and clinical healthcare—enabled the research team to draw out rich meaning (Wong et al. 2014; Greenhalgh 2026 (in press)).

We chose to focus the analysis on articles published between 2013 and 2023, as we found this period was when articles concerning advanced T1D proximal technologies such as sensors gained momentum. As this work was focused on understanding and enriching academic theorisation, we did not review grey literature, something that may be considered for further studies.

## 5 Conclusion

This hermeneutic review has shown the complexity of knowledge that people living with T1D have in relation to their proximal technologies. This knowledge encompasses clinical, technological and experiential knowledges, and these may coexist in multiple ways. Contrasting meta‐narratives help us illustrate different framings of this knowledge, which may be situated, emergent, distributed and embodied. Research to date is, however, still primarily undertaken in biomedical and related disciplines, which do not always capture the multiplicity of knowing in practice. We recommend that further research encompass both sociological and interdisciplinary approaches and includes greater use of theorisation informed by a ‘read‐across’ from different research traditions, which may include framings as yet unexplored by the literature. Such an approach may make a significant contribution to our understanding of how knowledge develops in the context of novel technologies, as self‐care through use of technology increases.

## Author Contributions

**Nadia Swann:** conceptualization, data curation, formal analysis, methodology, writing – original draft, writing – review and editing. **Jackie van Dael:** conceptualization, data curation, formal analysis, methodology, supervision, writing – original draft, writing – review and editing. **Henriette Langstrup:** writing – review and editing. **Eleanor Barry:** supervision, writing – review and editing. **Trisha Greenhalgh:** conceptualization, data curation, supervision, writing – review and editing. **Chrysanthi Papoutsi:** conceptualization, data curation, formal analysis, methodology, supervision, writing – original draft, writing – review and editing.

## Funding

The preparation of this review was supported by a UK National Institute of Health and Care Research School for Primary Care Research Capacity Award (C131 Nadia Swann) provided by the University of Oxford. The funders of this study had no role in study design, data collection, data analysis or data interpretation. The lead author had full access to all the data in the study and had final responsibility for the decision to submit for publication.

## Ethics Statement

The authors have nothing to report.

## Consent

The authors have nothing to report.

## Conflicts of Interest

Nadia Swann is a voluntary trustee director of Breakthrough T1D UK, a diabetes research charity. The authors declare that they have no other known competing financial interests or personal relationships that could have appeared to influence the work reported in this article.

## Supporting information


Supporting Information S1

## Data Availability

Qualitative data are available to research teams on reasonable request.
