# Supplementary material for: Framings of Patient Knowledge and Proximal Technologies in Type 1 Diabetes: A Hermeneutic Review Using Meta‐Narrative Methods
Source: Sociol Health Illn. 2026 Nov 1;48(8):e70287. doi: 10.1111/1467-9566.70287 (PMC13724230; doi:10.1111/1467-9566.70287)
Supplement: Supplementary file 1 [file SHIL-48-0-s001.docx]

**SUPPLEMENTAL INFORMATION APPENDIX**– Patient knowledge in the context of T1D proximal technologies

**Contents:**

- **Appendix A – Search terms and exclusion/inclusion criteria table**
- **Appendix B – Study characteristics tables**
- **Appendix C - Summary table of application of the 6 Guiding Principles from RAMESES training materials and Cochrane-Campbell Handbook**

**Appendix A -** **Search terms and exclusion/inclusion criteria table**

**Search terms table**

“type 1 diabet*” OR “type I diabet*” OR “T1D*” Or “t1d*”

AND know* OR attitude* OR exper* OR self-care OR self care OR self-management OR self management

AND technology OR wearable OR sensor OR biosensor OR biosensing device OR monitor* OR CGM OR pump OR closed-loop OR closed loop OR automated OR AID OR open-source OR open source OR DIY OR do-it-yourself OR apps OR applications

**Inclusion/Exclusion criteria table**

*Inclusion criteria* – studies on patient-facing proximal and related technologies for use primarily by patients, relatives or carers in type 1 diabetes care, patient and/or clinician experiences of patients, relatives or carers using type 1 diabetes technologies for patient care, all study designs and English language only

*Exclusion criteria* – studies on non-patient facing technologies, studies on conditions other than type 1 diabetes, studies describing technical components without discussing patient knowledge, experience, attitude or expertise, studies on distal T1D technologies not related to proximal technologies

**Appendix B – Study characteristics tables**

Study characteristics of 74 articles - table adapted from (Papoutsi *et al.*, 2021)

|  | **Name/**  **Authors** | **Year** | **Country** | **Type of paper** | **Theory used?** | **Study design/methods** | **Sample** | **Objectives** | **Research Tradition** |
| --- | --- | --- | --- | --- | --- | --- | --- | --- | --- |
| 1. 1 | (Abraham *et al.*, 2018) | 2018 | Australia | Research | No explicit  theorisation | Quantitative  ‘Case series’ approach  to self-management behaviours identified through pump and/or sensor data | 5 adolescent males with ‘deteriorating glycemic control’ | To identify and create awareness of ‘unexpected management behaviours’ and ‘to further explore the issue of trust and mistrust in medical technologies’ | **Biomedical** |
| 2. | (Addala *et al.*, 2021)* | 2021 | USA | Research | No explicit  theorisation | Quantitative  Survey | Bias assessment questionnaire ‘comprised of a clinical vignette and ranking exercises’ | To ‘determine if provider implicit bias’ ‘influences diabetes technology recommendation in the United States’ | **Biomedical** |
| 3. 2 | (Ahmed *et al.*, 2020) | 2020 | UK | Research | No explicit  theorisation | Qualitative  Ethnography | 2 patients  1 parent  1 healthcare professional | To present ‘perspectives of two patients, parents of a patient on discovering and using open-source automated insulin delivery and their healthcare provider’s perspective | **Biomedical** |
| 4. 3 | (Ardissone, 2021)* | 2021 | Italy | Research | Elias’ figurational and relational theories  Hirschman’s archetype (exit, voice and loyalty) | Qualitative  Semi-structured interviews | 67 interviews  47 patients and 20 diabetologists | To understand ‘how patients manage their satisfaction and dissatisfaction about the received care within interdependent figurations that are characterised by technology and intensive self-management requirements’ | **Sociomaterial**  **Knowledge studies/social learning** |
| 5. | (Ardissone, 2022)* | 2022 | Italy | Research | Actor-Network  Theory  Post-phenomenology | Qualitative  Interviews  Micro-  Case  studies | 6 participants | To explore the perspective of the user in why patients refuse to use a device, despite suggestions from diabetologists | **Sociomaterial** |
| 6. | (Asarani *et al.*, 2021) | 2021 | New Zealand | Review | No explicit theorisation | Literature  Review  Systematic  Review | 678 Articles  87 for full review  10 studies included | To conduct a systematic review of open-source closed-loop ‘efficacy, safety and user experience’ | **Biomedical** |
| 7. | (Barnard *et al.*, 2017)* | 2017 | UK Germany | Research | No explicit theorisation | Mixed  Methods  Questionnaires | 32 Adults | A psychosocial sub-study to ‘explore psychosocial experiences of closed loop technology’ | **Biomedical** |
| 8. 1 | (Beasant *et al.*, 2023) | 2023 | UK | Research | Normalisation Process Theory | Qualitative  Semi-structured interviews | 34 interviewees  10 young people  14 parents  10 HCPs | To explore  ‘experiences of using Libre and benefits and challenges to NHS | **Biomedical** |
| 9. | (Beneyto *et al.*, 2021)* | 2021 | Spain | Review | No explicit theorisation | Review and commentary | N/A | An analysis of potential faults in closed-loop introduced by users and ‘review of current fault tolerant control approaches’ to identify ‘missing areas of research’. | **Design** |
| 10. 4 | (Berk, 2018)* | 2018 | USA | Research | Barad’s theory of agency | Qualitative  Auto-ethnography  Journalling | Medical anthropologist analysing ‘personal medical experiences’ | To examine ‘pump-human entanglement’, and ‘how these subjectivities shift the lived experience of agency’ | **Sociomaterial** |
| 11. 5 | (Brew-Sam *et al.*, 2021)* | 2021 | Australia | Review | No explicit  theorisation | Systematic literature review and narrative synthesis | 528 references, 59 articles for full review, 17 articles included | ‘To describe the breadth of experiences and perspectives on diabetes technology use among children and adolescents with type 1 diabetes mellitus and their caregivers’ | **Biomedical** |
| 12. | (Burnside *et al.*, 2023)* | 2023 | New  Zealand | Research | Kaupapa  Maori  Research  Method-ologies  ‘participants positioned as experts’  Health Equity lens | Qualitative  Semi-structured interviews | 14 people | To explore ‘experiences of Indigenous Maoris participants in the CREATE Trial with use of an open-source’ system | **Biomedical** |
| 13. 6 | (Crabtree *et al.*, 2022)* | 2022 | UK | Research | No explicit  theorisation | Quantitative  Survey | 317 survey responses from a range of adult and paediatric healthcare professionals | To gain ‘insight’ into healthcare professionals’ ‘perceptions and practices around open-source automated insulin delivery’ | **Biomedical** |
| 14. 7 | (Cristello Sarteau *et al.*, 2023)* | 2023 | USA | Research | No explicit  theorisation | Qualitative  Focus groups | 29 older adults | To explore ‘experiences and treatment decisions among older adults, focusing on adopting care advances such as continuous glucose monitoring’ | **Biomedical** |
| 15. | (Crocket *et al.*, 2022) | 2022 | New  Zealand | Research | No explicit  theorisation | Qualitative  Thematic analysis  Semi-structured  interviews | 11 interviews with  13 parents  Participants from the Miao Miao Study | To ‘investigate the experiences of parents caring for young children with T1D using a DIY CGM in a supported setting | **Biomedical** |
| 16. 8 | (Danesi *et al.*, 2020)* | 2020 | Switz-  erland | Research | Theorisation of ‘knowledge-in-practice’ and ‘technology in practice’  References Mol and Law | Qualitative  Ethnographic observations of a 7 day summer camp  Workshop where attending young people and medical staff trialled a glucose sensor    Observation of consultations with diabetologist  Semi-structured interviews | 30 people aged 12-17    Patients and healthcare professionals living with T1D  27 semi-structured interviews with healthcare professionals 11 people with T1D and one with T2D and 3 parents | In relation to Flash/isCGM sensors,  to ‘explore how the users develop knowledge-in-practice’ | **Sociomaterial**  **Knowledge studies/social learning** |
| 17. | (DeMonaco *et al.*, 2019)* | 2019 | USA | Review | Consumer and lead user innovation theory | Commentary | N/A | To explore what happens when patients become innovators  What are the incentives that drive free patient innovation? | **Design**  Innovation |
| 18. 9 | (Elbalshy *et al.*, 2020) | 2020 | New Zealand | Research | No explicit  theorisation | Qualitative  Thematic analysis | 12 semi-structured interviews | To explore ‘experiences’ of families using DIY flash to continuous glucose monitoring and the ‘challenges’ | **Biomedical** |
| 19. 10 | (Farrington *et al.*, 2017) | 2017 | UK | Research | No explicit  Theorisation | Mixed methods  Surveys – Diabetes Technology  Questionnaire/  Hypoglycaemia Fear Survey  Braun and Clarke (2006) thematic analysis  (reference to Weick ‘Sense-making Framework)  Semi-structured interviews at beginning and end of study | 16 women | To ‘explore the experiences of pregnant women with type 1 diabetes to a ‘randomized crossover trial of sensor-augmented pump therapy vs automated closed-loop therapy’ | **Biomedical** |
| 20. 11 | (Farrington *et al.*, 2020)* | 2020 | UK | Research | No explicit  theorisation | Qualitative    Semi-structured interviews | 36 with healthcare professionals | To ‘explore clinician attitudes toward future mainstream closed-loop usage in England’ | **Biomedical** |
| 21. 12 | (François *et al.*, 2023)* | 2023 | France | Research | Self-Determination  Theory | Qualitative  Semi  structured  Interviews | 20 semi-structured interviews (13 women) | To ‘investigate whether technologies such as T1D sensors and pumps increase user empowerment’ | **Health**  **Psychology** |
| 22. | (Garmo *et al.*, 2013) | 2013 | Sweden | Research | No explicit  theorisation | Qualitative  Thematic  analysis  Interviews | 24 people  ‘saturation reached’ after 16 people | To ‘describe the experiences after 5 years of insulin pump use’ | **Biomedical** |
| 23. 7 | (Gonder-Frederick *et al.*, 2011) | 2011 | USA | Review | Health Belief  Model  Theory of  Planned Behaviour  Diffusion of Innovation  Theory | Narrative review and commentary | N/A | To ‘review psychological and behavioural factors that have influenced adoption and utilization of past technologies and ‘examine three theoretical frameworks’ relevant to ‘patient factors’ | **Health**  **Psychology**  **(Also Design Innovation)** |
| 24. 8 | (Gonder-Frederick *et al.*, 2016)* | 2016 | USA | Review | Health Belief  Model  Acceptance and Use of Technology Theory  Diffusion of Innovation  Theory | Narrative review and commentary | N/A | To consider practical and psychosocial limitations in the context of several technology-related theoretical frameworks which may influence patient adoption and continued use of technologies | **Health**  **Psychology**  **(Also Design**  **Innovation)** |
| 25. | (Gottlieb and Cluck, 2018)* | 2018 | USA | Research | Rabeharisoa – evidenced based activism  Kelty’s  recursive public | Qualitative  Interviews | 27 people including  parents, patient advocates and endocrinologists | To explore the ‘community of patients, caregivers and coders of open-source automated insulin delivery, which the authors call T1D ‘hackers’ | **Sociomaterial**  **Knowledge studies/social learning** |
| 26. 13 | (Hood and Duke, 2015) | 2015 | USA | Research | Arnott- developmental theory of emerging adulthood | Qualitative  ‘Hermeneutic design using van Manen phenomenological approach to lived experience’ | 9 people aged 19 -24 | To ‘investigate the meaning of living with an insulin pump for the management of type 1 diabetes during the period of emerging adulthood’ | **Health**  **Psychology** |
| 27. | (Hood *et al.*, 2022)* | 2022 | UK/  USA | Research | No explicit  theorisation | Mixed methods  Open label, single-period randomized parallel design  Surveys at baseline, 3/6 mths  Focus Group | 98 young people and their parents | To ‘examine changes in the lived experience of T1D after use of hybrid closed loop, including the Cam APS FX CL system’ | **Biomedical** |
| 28. | (Howe *et al.*, 2023)* | 2023 | USA | Research | No explicit  theorisation | Qualitative  Virtual  Focus Group  Use of Kilbourne Framework for racial disparities as a focus group guide | 21 parents | To ‘describe differences in non-Hispanic Black and non-Hispanic White patents’ perceptions of factors that influence the use of diabetes technology’ | **Biomedical** |
| 29. 14 | (Huhndt *et al.*, 2022)* | 2022 | Multi  national | Research | No explicit  theorisation | Quantitative  Survey  ‘Multinational, web-based cross-sectional study’ conducted with users and non-users of open-source automated insulin delivery’ | 1052 caregivers of children and adolescents under age 18 | To ‘investigate the barriers to scale-up open-source AID systems in caregivers of children and adolescents in the #WeAreNot  Waiting community’ | **Biomedical** |
| 30. 15 | (Jansky, 2023)* | 2023 | Germany | Research | Theories of ‘elaborative tinkering’ and ‘knowing in action’  References Mol and Pols  Theories of activism | Qualitative  Ethnography  Interviews | 28 patients/family  and healthcare professionals | To ‘explore knowledge practices’ in the “looper community” in type 1 diabetes | **Sociomaterial**  **Knowledge studies/social learning** |
| 31. 16 | (Jansky and Langstrup, 2023)* | 2023 | Germany | Research | Latourian ‘central matters of concern’  Theories of material participation  and device activism  Kelty’s  ‘recursive public’ | Qualitative  Ethnography  28 in-depth  interviews with ‘loopers, ex-loopers, their relatives and healthcare professionals’ | 28 people (12 women, 16 men) aged from 22 to 81. Six were healthcare professionals | To ‘explore the different forms of material participation encountered’ among ‘loopers’ | **Sociomaterial**  **Knowledge studies/social learning** |
| 32. | (Kaziunas *et al.*, 2017)* | 2017 | USA | Research | Lens of ‘caring-through -data’ | Qualitative  Ethnography | 21 semi-structured interviews with members of the Nightscout community | To ‘detail the experiences of parents using Nightscout’ | **Sociomaterial**  **Knowledge studies/social learning** |
| 33. 17 | (Kaziunas *et al.*, 2018)* | 2017 | USA | Research | Theories of ‘socio -technical tinkering’  References Mol and Haraway’s cyborg ontology | Qualitative  Ethnography | 21 semi-structured interviews including 11 parents, 9 of whom were Nightscout developers | To examine ‘two previously largely separated areas of research: (a) personal data, quantified self, and lived informatics and (b) DIY making and hacking ideas and practices’ using ‘DIY diabetes projects’ | **Sociomaterial**  **Knowledge studies/social learning** |
| 34. 18 | (Kesavadev *et al.*, 2020) | 2020 | India | Review | No explicit  theorisation | Narrative review and commentary | N/A | To provide ‘a comprehensive description of the DIYAPS – including their origin, existing literature, advantages, and disadvantages  To ‘enable physicians to make an independent decision on the use of DIYAPS’ | **Biomedical** |
| 35. 19 | (Kimbell *et al.*, 2020) | 2020 | UK | Research | No explicit  theorisation | Qualitative  Semi-structured interviews | 22 people – 7 doctors, 9 diabetes nurses and 6 research nurses | To ‘explore heath professionals’ views about the training support, and resourcing needed to support people using closed-loop technology in routine clinical care to help inform the development of formal guidance’ | **Biomedical** |
| 36. 7 | (Kubilay *et al.*, 2023)* | 2023 | Australia | Research | No explicit  theorisation | Qualitative  Semi-  Structured  Interviews | 21 older adults | To ‘explore the lived experience of older adults with T1D using closed-loop’ | **Biomedical**  **(Also Design**  **UX/Safety)** |
| 37. | (Lakshman *et al.*, 2024)* | 2024 | UK | Research | No explicit theorisation | Qualitative  Surveys  Interviews | 11 participants | A qualitative sub-study from the CLEAR trial which ‘ aimed to understand the psychosocial impact of using the fully automated system’ | **Biomedical** |
| 38. 20 | (Lawton *et al.*, 2016)* | 2016 | UK | Research | Grounded Theory | Qualitative  Interviews | 12 diabetic specialist nurses and 6 dieticians | To ‘explore health professionals’ views about insulin pump therapy’ and ‘the types of individuals they thought would gain greatest clinical benefit from using this treatment’ | **Biomedical** |
| 39. 21 | (Lawton *et al.*, 2019) | 2019 | UK | Research | Normalisation Process Theory approach – ‘recognising possible unintended consequ-ences arising from using a new technology’ | Qualitative  Semi-structured  interviews | 24 individuals  10 adults, 5 adolescents and 9 parents | To ‘understand and explore how people used the closed-loop to support diabetes management in order to aid interpretations of trial outcomes and provide recommendations for training and support with could be given to future users to help promote optimal use of the technology’ | **Biomedical** |
| 40. 22 | (Lawton *et al.*, 2020)* | 2020 | UK | Research | No explicit  theorisation | Qualitative  Interviews | 22 health care professionals | To ‘explore health professionals’ views about who would benefit from using a closed-loop system and who would be prioritized for access to the technology in routine clinical care’ | **Biomedical** |
| 41. 23 | (Lee *et al.*, 2017)* | 2017 | USA | Research | No explicit  theorisation | Quantitative  Survey | Survey completed by 1461 individuals | To ‘compare demographic/  disease characteristics of users versus non-users of Nightscout, ‘to describe its uses and personalization and to evaluate associated changes in health behaviours and outcomes | **Biomedical** |
| 42. 24 | (Luo *et al.*, 2022) | 2022 | China | Review | No explicit  theorisation | Literature  review  Systematic  Review and meta synthesis of qualitative studies | 945 articles, 58 articles for review  Nine studies met the inclusion criteria | To explore the experiences and perspectives of the combined use ‘of pumps and sensors on parents of children with T1D’ | **Biomedical** |
| 43. 8 | (Manero, 2023) | 2023 | USA | Research | Theory of self  management  /mastery | Qualitative  Semi-  Structured  Interviews  Fearon-Lynch used in creating interview questions | 11 adults | To ‘explore the perspectives and experiences of adults with T1D’ using closed-loop | **Biomedical** |
| 44. 25 | (Marks *et al.*, 2019) | 2019 | USA | Research | No explicit  theorisation | Quantitative  Survey | 86 surveys received from 42 pediatric endocrinology fellows and 69 attending physicians in pediatric endocrinology | To ‘assess fellows’ knowledge, attitudes, and practices regarding insulin pumps and CGMs’ | **Biomedical** |
| 45. 26 | (Marshall *et al.*, 2019)* | 2019 | UK | Research | No explicit  theorisation | Qualitative  Ethnographic | 2 adults  1 caregiver  3 healthcare professionals | To give perspectives of 2 adult users, a caregiver a of learning to build and use a ‘do-it-yourself artificial pancreas and 3 physicians’ responses | **Biomedical** |
| 1. 1 46. | (Mathieu-Fritz and Guillot, 2017)* | 2017 | France | Research | Strauss -theory of patient work | Qualitative  Semi structured interviews  Technology ‘show and tell’  Survey | 17 patients  1 diabetologist  50 survey responses | To’ describe the specific effects of the use of ‘diabetes self-monitoring systems’ on ‘patient work’ and the illness experience | **Sociomaterial**  **Knowledge studies/social learning** |
| 47. 27 | (McCarthy *et al.*, 2021)* | 2021 | New Zealand | Research | Grounded theory | Qualitative  Ethnographic case study  Cultural probes and letter writing | 9 participants aged 12-24  3 male and 6 female | To ‘trial a method of writing love letters and break-up letters to medical devices’ in order to uncover adolescents wide-ranging self-management experiences and psychosocial user requirements’ | **Design**  **(UX/Safety)** |
| 48. 29 | (Mialet, 2022)* | 2022 | USA | Research | Distributed cognition theory | Qualitative  Ethnography  Auto-  ethnography | Interviews | To ‘explore through the lens of type 1 diabetes what a body in fluctuation feels’ and to ‘investigate the complexity of relying on sensations that appear or disappear, on other beings that have their own agenda, or on machines that could help or kill’ | **Sociomaterial**  **Knowledge studies/social learning** |
| 49. 9 | (Morrow *et al.*, 2022)* | 2022 | UK | Research | No explicit  theorisation | Qualitative  Thematic analysis  Photovoice methods – Photos with reflective text | 8 dyads – parent and adolescent  5 parents individually  8 adolescents individually | To ‘explore how technologies and physical activity are experienced by adolescents with T1D’ | **Design**  **(UX/Safety)** |
| 50. 30 | (Murray *et al.*, 2019) | 2019 | USA | Research | No explicit  theorisation | Mixed methods  Survey  Observational study  2 phases – phase 1 to address HCP technology experience and knowledge and phase 2 to evaluate an educational factsheet. | Participants were 43 attendees at an American Association of Diabetes Educators (AADE) conference for phase 1 and 137 members of AADE and Diabetes Advanced Network Access (DANA) for phase 2 | To ‘assess HCP technology experience, AID knowledgebase, comfort levels answering patient questions’ about open-source AID technology, and ‘desire for further education’ | **Biomedical** |
| 51. 31 | (Natale *et al.*, 2023)* | 2023 | Australia | Review | No explicit  theorisaton | Literature  review  Systematic review of qualitative studies relating to continuous glucose monitors | 9444 citations  446 full text reviews  54 studies included | To review patient  experiences of CGMs  and pumps | **Biomedical** |
| 52. 10 | (Nefs, 2022)* | 2022 | Netherlands | Review | No explicit  theorisation | Narrative  review and commentary | N/A | Overview of the  psychological implications of automated insulin delivery to understand ‘person-technology interactions’ | **Health**  **Psychology** |
| 53. 32 | (Ng *et al.*, 2020)* | 2020 | USA | Review | References to lead-user theory | Qualitative  Review of social media sites  Thematic analysis | Review of communications on CGM in the Cloud Facebook group and coding site GitHub | To ‘thematically analyse ‘the evolution of patient-driven innovations from the NightScout project | **Biomedical**  **(Also Design/**  **Innovation)** |
| 54. 34 | (O'Donnell *et al.*, 2023)* | 2023 | Multi-  national | Research | No explicit  theorisation | Quantitative  Questionnaire sent to non-users | 129 participants from 31 countries including Canada, Germany, Ireland, UK and USA | To ‘investigate the perceived barriers towards adoption and maintaining of open-source systems’ | **Biomedical** |
| 55. | (Øversveen, 2020) | 2020 | Norway | Research | Latour  Akrich  Hutchby - affordance theory | Qualitative  Theorisation study based on qualitative interview data from in-depth interviews | 24 people | To theorise health inequalities through ‘social constructivist perspectives on user-technology relations’ | **Sociomaterial**  **Knowledge studies/social learning** |
| 56. 35 | (Øversveen, 2021)* | 2021 | Norway | Research | Fundamental cause theory  Compensation inversion theory  Patient empowerment theories | Qualitative  case study  Interviews and observations | 8 healthcare professionals  24 patients  20 staff meetings observed | To ‘examine how medical staff with to distribute limited technological resources among patients’ | **Sociological** |
| 57. 36 | (Palmer *et al.*, 2020) | 2020 | USA | Research | No explicit  theorisation | Quantitative  Survey of patients and providers relating to patients’ use of open-source automated insulin delivery | 101 patients  152 providers | To ‘assess perspectives of US patients and diabetes providers on the safety and efficacy’ of open-source automated insulin delivery,  how ‘providers are approaching this technology’ and ‘how the internet-based peer support network has impacted the patient-provider relationship’ | **Biomedical** |
| 58. 37 | (Perez *et al.*, 2019)* | 2019 | USA | Research | Uncertainty management theory | Qualitative  Interviews  Thematic analysis – Braun and Clarke 2006 | 29 participants | To explore ‘what types of uncertainty to parents of a child with T1D report experiencing’ and ‘how do parents manage this uncertainty’? | **Health Psychology** |
| 59. 38 | (Raimond *et al.*, 2022)* | 2022 | Denmark | Research | No explicit  theorisation | Qualitative  Semi-structured interviews  Thematic analysis | 5 patient participants  6 healthcare professionals who had more than 2 years’ experience with insulin pump therapy | To ‘explore the challenges that open-source automated insulin delivery systems pose to the encounter between service-users and healthcare professionals’ | **Biomedical** |
| 60. 13 | (Rankin *et al.*, 2022)* | 2022 | UK | Research | Grounded  theory | Qualitative  Thematic  analysis | 18 adolescents  21 parents | To ‘explore adolescents’ and parents’ experiences of suing a closed-loop system to understand how it affects their biographies and everyday lives’ | **Biomedical** |
| 61. 39 | (Rankin *et al.*, 2023) | 2023 | UK | Research | No explicit  theorisation | Qualitative  ‘descriptive analysis’  Semi structured interviews | 19 healthcare professionals involved in the AiDAPT trial (automated insulin delivery amongst pregnant women) | To ‘explore healthcare professionals’ views about the training and support needed to rollout closed-loop technology to pregnant women with type 1 diabetes’ | **Biomedical** |
| 62. 40 | (Reidy *et al.*, 2018) | 2018 | UK | Review | No explicit  theorisation | Literature review  Critical interpretative synthesis | 4998 titles,  274 abstracts reviewed, 39 full articles retrieved and 22 analysed | To ‘determine what influences the incorporation, adaptation and use’ of pump therapy in the ‘everyday lives’ of people with T1D | **Health**  **Psychology** |
| 63. | (Reinius *et al.*, 2022)* | 2022 | Sweden | Review | No explicit  Theorisation | Literature  review and commentary | N/A | What is the nature and extent of patient-driven innovations published in peer-reviewed scientific journals? | **Design**  Innovation |
| 64. 41 | (Schipp *et al.*, 2021) | 2021 | Australia | Research | No explicit  theorisation | Qualitative  Semi-structured interviews | 23 participants | To explore the ‘experiences’ of adults with T1D’ using open-source automated insulin delivery ‘to understand how they are navigating these challenges’ | **Biomedical** |
| 65. 42 | (Sehgal *et al.*, 2023) | 2023 | New Zealand | Research | No explicit  theorisation | Qualitative  Questionnaire and semi-structured interviews | 12 people | To ‘explore user experiences with DIY CGM in people aged 16-69 years’ with T1D | **Biomedical** |
| 66. 43 | (Staszak *et al.*, 2021)* | 2021 | Germany | Review | No explicit  theorisation | Quantitative  Metrics used to assess whether patients’ use of custom DIY features improved outcomes  Metrics calculated from donated CGM data of users | N/A | To ‘expand the existing knowledge’ on open-source automated insulin delivery’ which has been ‘gaining popularity among the patient population’ | **Design**  **(UX/Safety)** |
| 67. 44 | (Suttiratana *et al.*, 2022)* | 2022 | USA | Research | No explicit  theorisation | Qualitative  Focus groups | 72 users  new and experienced users and 19 discontinuers recruited to focus groups | To ‘assess multi-dimensional aspects of Loop user experiences’ | **Design**  **(UX/Safety)** |
| 68. | (Tanenbaum *et al.*, 2017)* | 2017 | USA | Research | No explicit  theorisation | Quantitative  Survey | Survey of 209 clinicians | To ‘examine perceptions of barriers to device uptake, attitudes towards diabetes technology and resources needed for clinicians to improve device uptake’ | **Biomedical** |
| 69. 45 | (Tanenbaum *et al.*, 2020) | 2020 | USA | Research | Diffusion of Innovation  Technology Acceptance Model | Qualitative  Focus groups | Participants recruited from a ‘multicenter trial’ of hybrid closed loop systems (Ly et al 2016) | To explore ‘what factors influence trust in HCL systems’ and how users ‘experience giving over control to an automated system’ | **Health**  **Psychology**  **(Also Design**  **UX/Safety)** |
| 70. | (Tanenbaum *et al.*, 2021a) | 2021 | USA | Research | No explicit theorisation | Qualitative  Focus Groups | 22 participants | To explore preferences that adults with T1D have for “training and support to initiate and sustain” sensor use | **Biomedical** |
| 71. 11 | (Tanenbaum *et al.*, 2021b) | 2021 | USA | Research | No explicit  theorisation | Qualitative  Focus groups  Semi-structured  Interviews | 16 parents | ‘Parent experiences with early introduction of CGM following their child’s T1D diagnosis’ | **Biomedical** |
| 72. | (Tanenbaum and Commissariat, 2023)* | 2023 | USA | Review | No explicit theorisation | Narrative review and commentary | N/A | To “describe personal challenges and reactions that users experience when interacting with current diabetes technologies” | **Biomedical** |
| 73. 46 | (Todres *et al.*, 2010) | 2010 | UK | Research | No explicit  theorisation | Qualitative  Ethnography  ‘phenomenological design’ | 4 patients with ‘varied experience of pump use’ | To ‘provide in-depth insight into the changes that may be experienced by patients embarking on continuous subcutaneous insulin infusion’ and ‘what is it like to live with an insulin pump’ | **Biomedical** |
| 74. | (Tsai *et al.*, 2022)* | 2022 | USA | Research | No explicit  theorisation | Quantitative  Surveys  Technology Use  Attitudes and  Barriers to Device Use  Question-  naires | 173 people  30 Spanish speaking  143 English speaking | To explored potential differences between Latinx and non-Latinx Spanish/English speaking patients in ‘attitudes about and barriers to diabetes technologies’ | **Biomedical** |

**Appendix C - Summary table of application of the 6 Guiding Principles from RAMESES training materials (Wong et al., 2014) and Cochrane-Campbell Handbook incorporating meta-narrative methodology (Noyes et al., April 2025 )**

| **Principle:** | **Summary**: | **Application of principles to review** |
| --- | --- | --- |
| **Pragmatism** | Review should focus on what is useful; namely what is “likely to promote sensemaking” | The lead researcher’s expertise and background as a director of a T1D medical research charity, a parent, sibling and niece of people living with T1D, and as an academic researcher was instrumental in eliciting meaningful contributions from academic literature likely to ‘promote sensemaking’, while remaining mindful of the researcher’s own particular viewpoints and assumptions.  See also **Peer Review** below. |
| **Pluralism** | Review should “develop an account of the topic area that is illuminated from multiple angles and perspectives” | The review follows the principle of pluralism by approaching the topic through the lenses of different research traditions, and through tracing and giving an account of each narrative, while remaining mindful of the background described above in considering perspectives of clinicians, design engineers and others. |
| **Historicity or**  **Temporality** | Research traditions “are often best described as they unfolded over time” | The review considered the origins of each research tradition to explore and show how views of patient knowledge of T1D proximal technologies may have developed over time. |
| **Contestation** | “Conflicting data” from different research traditions should be examined to generate “higher order insights” (including how different research teams framed the issue differently) | The review includes a discussion section which considers tensions and differences in approach between research traditions, considers possible explanations for these differences and produces insights regarding theorisation approaches. |
| **Reflexivity** | Need to “continually reflect, individually and as a team” on emerging findings | See above on **Pragmatism** and section on reflections/reflexivity in review regarding refinement and focusing of approaches. |
| **Peer Review** | “Emerging findings should be presented to an external audience” alongside reflexivity through peer review to avoid the dangers of being a single researcher “flying solo” | See **Pragmatism** above.  While the lead reviewer was responsible for selection and first analysis of the articles based on both her academic and personal experience of the topic, an experienced academic colleague provided close in-depth reading of significant articles to contribute to the process. Peer review also included an immersive process of sensemaking and interpretation process across a highly experienced six-person research team to ensure rigour of approach. |

Noyes, J.*, et al.* ( April 2025 (draft)) 'Chapter 19 Introducing meta-narrative reviews, critical interpretative synthesis and meta-aggregation ', in Noyes J, H.A. (ed.) *Cochrane-Campbell Handbook for Qualitative Evidence Synthesis, Version 1*. London: Cochrane.

Wong, G.*, et al.* (2014) 'Development of methodological guidance, publication standards and training materials for realist and meta-narrative reviews: the RAMESES (Realist And Meta-narrative Evidence Syntheses – Evolving Standards) project', *Health Serv Deliv Res,* 2.

**References:**

Abraham, M. B., et al. (2018) 'Unexpected Management Behaviors in Adolescents With Type 1 Diabetes Using Sensor-Augmented Pump Therapy', *Journal of Diabetes Science and Technology,* 12(3), pp. 592-598.

Addala, A., et al. (2021) 'Provider Implicit Bias Impacts Pediatric Type 1 Diabetes Technology Recommendations in the United States: Findings from The Gatekeeper Study', *Journal of Diabetes Science and Technology,* 15(5), pp. 1027-1033.

Ahmed, S. H., et al. (2020) 'Do-It-Yourself (DIY) Artificial Pancreas Systems for Type 1 Diabetes: Perspectives of Two Adult Users, Parent of a User and Healthcare Professionals', *Advances in Therapy,* 37(9), pp. 3929-3941.

Ardissone, A. (2021) 'From loyalty to resignation: Patient-doctor figurations in type 1 diabetes', *SOCIOLOGY OF HEALTH & ILLNESS,* 43(6), pp. 1388-1404.

Ardissone, A. (2022) 'Selective adoption of therapeutic devices among people with type 1 diabetes', *Health Sociology Review,* 31(3), pp. 278-292.

Asarani, N. A. M., et al. (2021) 'Efficacy, safety, and user experience of DIY or open-source artificial pancreas systems: a systematic review', *Acta Diabetologica,* 58(5), pp. 539-547.

Barnard, K. D., et al. (2017) 'Closing the Loop in Adults, Children and Adolescents With Suboptimally Controlled Type 1 Diabetes Under Free Living Conditions: A Psychosocial Substudy', *Journal of Diabetes Science and Technology,* 11(6), pp. 1080-1088.

Beasant, L., et al. (2023) 'Flash glucose monitoring in young people with type 1 diabetes—a qualitative study of young people, parents and health professionals: ‘ *It makes life much easier’*', *BMJ Open,* 13(4), pp. e070477.

Beneyto, A., et al. (2021) 'Fault Tolerant Strategies for Automated Insulin Delivery Considering the Human Component: Current and Future Perspectives', *Journal of Diabetes Science and Technology,* 15(6), pp. 1224-1231.

Berk, E. (2018) 'A kind of disassembled and reassembled, postmodern collective and personal self: Agency and the Insulin pump', *Journal of Material Culture,* 23(4), pp. 448-458.

Brew-Sam, N., et al. (2021) 'Experiences of Young People and Their Caregivers of Using Technology to Manage Type 1 Diabetes Mellitus: Systematic Literature Review and Narrative Synthesis', *JMIR Diabetes,* 6(1), pp. e20973.

Burnside, M., et al. (2023) 'Interviews with Indigenous Māori with type 1 diabetes using open-source automated insulin delivery in the CREATE randomised trial', *Journal of Diabetes & Metabolic Disorders,* 22(1), pp. 861-871.

Crabtree, T. S. J., et al. (2022) 'Association of British Clinical Diabetologists, Diabetes Technology Network UK and Association of Children’s Diabetes Clinicians Survey of UK Healthcare Professional Attitudes Towards Open-Source Automated Insulin Delivery Systems', *Diabetes Therapy,* 13(2), pp. 341-353.

Cristello Sarteau, A., et al. (2023) 'Supporting the 'lived expertise' of older adults with type 1 diabetes: An applied focus group analysis to characterize barriers, facilitators, and strategies for self-management in a growing and understudied population', *Diabetic Medicine: A Journal of the British Diabetic Association*, pp. e15156.

Crocket, H., et al. (2022) 'Parental experiences of short term supported use of a do-it-yourself continuous glucose monitor (DIYrtCGM): A qualitative study', *Diabetic Medicine,* 39(5), pp. e14731.

Danesi, G., et al. (2020) 'Techno-social reconfigurations in diabetes (self-) care', *Social Studies of Science,* 50(2), pp. 198-220.

DeMonaco, H. J., et al. (2019) 'When Patients Become Innovators', *MIT Sloan Management Review, Spring*.

Elbalshy, M., et al. (2020) 'Exploring Parental Experiences of Using a Do-It-Yourself Solution for Continuous Glucose Monitoring Among Children and Adolescents With Type 1 Diabetes: A Qualitative Study', *Journal of Diabetes Science and Technology,* 14(5), pp. 844-853.

Farrington, C., et al. (2020) 'Who Should Access Closed-Loop Technology? A Qualitative Study of Clinician Attitudes in England', *Diabetes Technology & Therapeutics,* 22(5), pp. 404-410.

Farrington, C., et al. (2017) 'Experiences of closed‐loop insulin delivery among pregnant women with Type 1 diabetes', *Diabetic Medicine,* 34(10), pp. 1461-1469.

François, J., et al. (2023) 'Empowering health care consumers in the era of Internet of Things', *International Journal of Consumer Studies,* 47(3), pp. 1060-1075.

Garmo, A., et al. (2013) '‘The pump was a saviour for me.’Patients' experiences of insulin pump therapy', *Diabetic Medicine,* 30(6), pp. 717-723.

Gonder-Frederick, L., et al. (2011) 'Closed-loop glucose control: psychological and behavioral considerations', *Journal of Diabetes Science and Technology,* 5(6), pp. 1387-1395.

Gonder-Frederick, L. A., et al. (2016) 'Psychology, technology, and diabetes management', *American Psychologist,* 71(7), pp. 577-589.

Gottlieb, S. D. and Cluck, J. (2018) '“Going Rogue” re-coding resistance with type 1 diabetes', *Digital Culture & Society,* 4(2), pp. 137-156.

Hood, et al. (2022) 'Lived experience of CamAPS FX closed loop system in youth with type 1 diabetes and their parents', *Diabetes, Obesity & Metabolism,* 24(12), pp. 2309-2318.

Hood, D. G. and Duke, G. (2015) 'The Nature and Meaning of Insulin Pump Use in Emerging Adults With Type 1 Diabetes', *Diabetes Spectrum,* 28(2), pp. 75-81.

Howe, C. J., et al. (2023) 'Racial Disparities in Technology Use in Children With Type 1 Diabetes: A Qualitative Content Analysis of Parents' Perspectives', *The Science of Diabetes Self-Management and Care,* 49(1), pp. 55-64.

Huhndt, A., et al. (2022) 'Barriers to Uptake of Open-Source Automated Insulin Delivery Systems: Analysis of Socioeconomic Factors and Perceived Challenges of Caregivers of Children and Adolescents With Type 1 Diabetes From the OPEN Survey', *Frontiers in Clinical Diabetes and Healthcare,* 3, pp. 876511.

Jansky, B. (2023) 'Digitized Patients: Elaborative Tinkering and Knowledge Practices in the Open-source Type 1 Diabetes “Looper Community”', *Science, Technology, & Human Values*, pp. 01622439231170443.

Jansky, B. and Langstrup, H. (2023) 'Device activism and material participation in healthcare: retracing forms of engagement in the #WeAreNotWaiting movement for open-source closed-loop systems in type 1 diabetes self-care', *BioSocieties,* 18(3), pp. 498-522.

Kaziunas, E., et al. 'Caring through Data: Attending to the Social and Emotional Experiences of Health Datafication'. *CSCW '17*. February 25, 2017. New York, NY, USA: Association for Computing Machinery, 2260–2272.

Kaziunas, E., et al. (2018) 'Lived Data: Tinkering With Bodies, Code, and Care Work', *Human–Computer Interaction,* 33(1), pp. 49-92.

Kesavadev, J., et al. (2020) 'The Do-It-Yourself Artificial Pancreas: A Comprehensive Review', *Diabetes Therapy,* 11(6), pp. 1217-1235.

Kimbell, B., et al. (2020) 'What Training, Support, and Resourcing Do Health Professionals Need to Support People Using a Closed-Loop System? A Qualitative Interview Study with Health Professionals Involved in the Closed Loop from Onset in Type 1 Diabetes (CLOuD) Trial', *Diabetes Technology & Therapeutics,* 22(6), pp. 468-475.

Kubilay, E., et al. (2023) 'Lived experience of older adults with type 1 diabetes using closed-loop automated insulin delivery in a randomised trial', *Diabetic Medicine,* 40(4), pp. e15020.

Lakshman, R., et al. (2024) 'Lived Experience of Fully Closed-Loop Insulin Delivery in Adults with Type 1 Diabetes', *Diabetes Technology & Therapeutics,* 26(4), pp. 211.

Lawton, J., et al. (2019) 'Ambassadors of hope, research pioneers and agents of change—individuals’ expectations and experiences of taking part in a randomised trial of an innovative health technology: longitudinal qualitative study', *Trials,* 20, pp. 1-12.

Lawton, J., et al. (2020) 'Health professionals' views about who would benefit from using a closed-loop system: a qualitative study', *Diabetic Medicine,* 37(6), pp. 1030-1037.

Lawton, J., et al. (2016) 'Who gains clinical benefit from using insulin pump therapy? A qualitative study of the perceptions and views of health professionals involved in the Relative Effectiveness of Pumps over MDI and Structured Education (REPOSE) trial', *Diabetic Medicine: A Journal of the British Diabetic Association,* 33(2), pp. 243-251.

Lee, J. M., et al. (2017) 'Real-World Use and Self-Reported Health Outcomes of a Patient-Designed Do-it-Yourself Mobile Technology System for Diabetes: Lessons for Mobile Health', *Diabetes Technology & Therapeutics,* 19(4), pp. 209-219.

Luo, X., et al. (2022) 'Parents' experiences on the combined use of continuous subcutaneous insulin infusion and real‐time continuous glucose monitoring to manage Type 1 diabetes in their children: A systematic review and meta‐synthesis of qualitative studies', *Nursing Open,* 9(6), pp. 2532.

Manero, C. (2023) 'Experiences of Patients Adopting and Adapting to Closed-Loop Insulin Delivery Systems (CLIDS)', *SCIENCE OF DIABETES SELF-MANAGEMENT AND CARE,* 49(1), pp. 46-54.

Marks, B. E., et al. (2019) 'Pediatric Endocrinology Trainees' Education and Knowledge About Insulin Pumps and Continuous Glucose Monitors', *Diabetes Technology & Therapeutics,* 21(3), pp. 105-109.

Marshall, D. C., et al. (2019) 'Do-It-Yourself Artificial Pancreas Systems in Type 1 Diabetes: Perspectives of Two Adult Users, a Caregiver and Three Physicians', *Diabetes Therapy,* 10(5), pp. 1553-1564.

Mathieu-Fritz, A. and Guillot, C. (2017) 'Diabetes self-monitoring devices and transformations in “patient work”', *Revue d’anthropologie des connaissances,* 11(4).

McCarthy, G. M., et al. (2021) 'Letters to medical devices: a case study on the medical device user requirements of adolescents with type 1 diabetes', *BEHAVIOUR & INFORMATION TECHNOLOGY,* 40(1), pp. 39-48.

Mialet, H. (2022) 'Bodies in Balance: Tracking Type 1 Diabetes', *Body and Society,* 28(3), pp. 89–113.

Morrow, D., et al. (2022) 'Letting the World See through Your Eyes: Using Photovoice to Explore the Role of Technology in Physical Activity for Adolescents Living with Type 1 Diabetes', *International Journal of Environmental Research and Public Health,* 19(10), pp. 6315.

Murray, J. A., et al. (2019) 'Health Care Provider Knowledge and Perceptions of FDA-Approved and Do-It-Yourself Automated Insulin Delivery', *Journal of Diabetes Science and Technology,* 14(6), pp. 1017-1021.

Natale, P., et al. (2023) 'Patient experiences of continuous glucose monitoring and sensor-augmented insulin pump therapy for diabetes: A systematic review of qualitative studies', *Journal of Diabetes,* 15(12), pp. 1048-1069.

Nefs, G. (2022) 'The Psychological Implications of Automated Insulin Delivery Systems in Type 1 Diabetes Care', *Frontiers in Clinical Diabetes and Healthcare,* 3, pp. 846162.

Ng, M., et al. (2020) 'Evolution of Do-It-Yourself Remote Monitoring Technology for Type 1 Diabetes', *Journal of Diabetes Science and Technology,* 14(5), pp. 854-859.

Noyes, J., et al. ( April 2025 ) 'Chapter 19 Introducing meta-narrative reviews, critical interpretative synthesis and meta-aggregation (draft April 2025)', in Noyes J, H.A. (ed.) *Cochrane-Campbell Handbook for Qualitative Evidence Synthesis, Version 1*. London: Cochrane.

O'Donnell, S., et al. (2023) 'Barriers to uptake of Open-Source automated insulin delivery Systems: Analysis of socioeconomic factors and perceived challenges of adults with type 1 diabetes from the OPEN survey', *Diabetes Research and Clinical Practice,* 197, pp. 110235.

Øversveen, E. (2020) 'Stratified users and technologies of empowerment: theorising social inequalities in the use and perception of diabetes self-management technologies', *Sociology of Health & Illness,* 42(4), pp. 862-876.

Øversveen, E. (2021) 'Need, competence and compliance: Selective empowering in the distribution of medical technologies in the Norwegian health care system', *Acta Sociologica,* 64(2), pp. 184-197.

Palmer, W., et al. (2020) 'Using a Do-It-Yourself Artificial Pancreas: Perspectives from Patients and Diabetes Providers', *Journal of Diabetes Science and Technology,* 14(5), pp. 860-867.

Papoutsi, C., et al. (2021) 'Interrogating the promise of technology in epilepsy care: systematic, hermeneutic review', *Sociology of Health & Illness,* 43(4), pp. 928-947.

Perez, L., et al. (2019) 'Communicatively Exploring Uncertainty Management of Parents of Children with Type 1 Diabetes', *Health Communication,* 34(9), pp. 949-957.

Raimond, L. H., et al. (2022) 'Open-source automated insulin delivery systems and formal healthcare: A qualitative study of challenges in the interaction between service-users with type 1 diabetes and healthcare professionals', *Chronic Illness,* 19(4), pp. 836-847.

Rankin, D., et al. (2023) 'Rollout of Closed-Loop Technology to Pregnant Women with Type 1 Diabetes: Healthcare Professionals' Views About Potential Challenges and Solutions', *Diabetes Technology & Therapeutics,* 25(4), pp. 260-269.

Rankin, D., et al. (2022) 'Adolescents’ and their parents’ experiences of using a closed-loop system to manage type 1 diabetes in everyday life: qualitative study', *Chronic Illness,* 18(4), pp. 742-756.

Reidy, C., et al. (2018) 'The process of incorporating insulin pumps into the everyday lives of people with Type 1 diabetes: A critical interpretive synthesis', *Health Expectations,* 21(4), pp. 714-729.

Reinius, M., et al. (2022) 'Patient-driven innovations reported in peer-reviewed journals: a scoping review', *BMJ Open,* 12(1), pp. e053735.

Schipp, J., et al. (2021) 'How Adults with Type 1 Diabetes Are Navigating the Challenges of Open-Source Artificial Pancreas Systems: A Qualitative Study', *Diabetes Technology & Therapeutics,* 23(8), pp. 546-554.

Sehgal, S., et al. (2023) 'Do-it-yourself continuous glucose monitoring in people aged 16 to 69 years with type 1 diabetes: A qualitative study', *Diabetic Medicine: A Journal of the British Diabetic Association*, pp. e15168.

Staszak, W., et al. 'Impact of Custom Features of Do-it-yourself Artificial Pancreas Systems (DIYAPS) on Glycemic Outcomes of People with Type 1 Diabetes'. *2021 43rd Annual International Conference of the IEEE Engineering in Medicine & Biology Society (EMBC)*, 2021-11, 1472-1475.

Suttiratana, S. C., et al. (2022) 'Qualitative Study of User Experiences with Loop, an Open-Source Automated Insulin Delivery System', *Diabetes Technology & Therapeutics,* 24(6), pp. 416-423.

Tanenbaum, M. L., et al. (2017) 'Optimal Use of Diabetes Devices: Clinician Perspectives on Barriers and Adherence to Device Use', *Journal of Diabetes Science and Technology,* 11(3), pp. 484-492.

Tanenbaum, M. L. and Commissariat, P. V. (2023) 'Experience with burdens of diabetes device use that affect uptake and optimal use in people with type 1 diabetes', *Endocrine Connections,* 12(10), pp. e230193.

Tanenbaum, M. L., et al. (2020) 'Trust in hybrid closed loop among people with diabetes: Perspectives of experienced system users', *Journal of Health Psychology,* 25(4), pp. 429-438.

Tanenbaum, M. L., et al. (2021a) 'Help when you need it: Perspectives of adults with T1D on the support and training they would have wanted when starting CGM', *Diabetes research and clinical practice,* 180, pp. 109048.

Tanenbaum, M. L., et al. (2021b) '‘I was ready for it at the beginning’: Parent experiences with early introduction of continuous glucose monitoring following their child's Type 1 diabetes diagnosis', *Diabetic Medicine,* 38(8), pp. e14567.

Todres, L., et al. (2010) 'Continuous subcutaneous insulin infusion in Type 1 diabetes: patient experiences of 'living with a machine'', *Diabetic Medicine: A Journal of the British Diabetic Association,* 27(10), pp. 1201-1204.

Tsai, D., et al. (2022) 'Diabetes Technology Experiences Among Latinx and Non-Latinx Youth with Type 1 Diabetes', *Journal of Diabetes Science and Technology,* 16(4), pp. 834-843.

Wong, G., et al. (2014) 'Development of methodological guidance, publication standards and training materials for realist and meta-narrative reviews: the RAMESES (Realist And Meta-narrative Evidence Syntheses – Evolving Standards) project', *Health Serv Deliv Res,* 2.
